# A multi-step machine learning approach to assess the impact of COVID-19 lockdown on NO_2_ attributable deaths in Milan and Rome, Italy

**DOI:** 10.1186/s12940-021-00825-9

**Published:** 2022-01-16

**Authors:** Luca Boniardi, Federica Nobile, Massimo Stafoggia, Paola Michelozzi, Carla Ancona

**Affiliations:** 1 Department of Epidemiology, Lazio Regional Health Service/ASL, Roma 1, Via C. Colombo 112, 00147 Rome, Italy; 2 EPIGET - Epidemiology, Epigenetics, and Toxicology Lab, Department of Clinical Sciences and Community Health, University of Milan, Milan, Italy; 3 Fondazione IRCCS Ca’ Granda Ospedale Maggiore Policlinico, Environmental and Industrial Toxicology Unit, Milan, Italy

**Keywords:** Air pollution, Health Impact Assessment, COVID-19, Citizen science, Machine Learning

## Abstract

**Background:**

Air pollution is one of the main concerns for the health of European citizens, and cities are currently striving to accomplish EU air pollution regulation. The 2020 COVID-19 lockdown measures can be seen as an unintended but effective experiment to assess the impact of traffic restriction policies on air pollution. Our objective was to estimate the impact of the lockdown measures on NO_2_ concentrations and health in the two largest Italian cities.

**Methods:**

NO_2_ concentration datasets were built using data deriving from a 1-month citizen science monitoring campaign that took place in Milan and Rome just before the Italian lockdown period. Annual mean NO_2_ concentrations were estimated for a lockdown scenario (Scenario 1) and a scenario without lockdown (Scenario 2), by applying city-specific annual adjustment factors to the 1-month data. The latter were estimated deriving data from Air Quality Network stations and by applying a machine learning approach. NO_2_ spatial distribution was estimated at a neighbourhood scale by applying Land Use Random Forest models for the two scenarios. Finally, the impact of lockdown on health was estimated by subtracting attributable deaths for Scenario 1 and those for Scenario 2, both estimated by applying literature-based dose–response function on the counterfactual concentrations of 10 μg/m^3^.

**Results:**

The Land Use Random Forest models were able to capture 41–42% of the total NO_2_ variability. Passing from Scenario 2 (annual NO_2_ without lockdown) to Scenario 1 (annual NO_2_ with lockdown), the population-weighted exposure to NO_2_ for Milan and Rome decreased by 15.1% and 15.3% on an annual basis. Considering the 10 μg/m^3^ counterfactual, prevented deaths were respectively 213 and 604.

**Conclusions:**

Our results show that the lockdown had a beneficial impact on air quality and human health. However, compliance with the current EU legal limit is not enough to avoid a high number of NO_2_ attributable deaths. This contribution reaffirms the potentiality of the citizen science approach and calls for more ambitious traffic calming policies and a re-evaluation of the legal annual limit value for NO_2_ for the protection of human health.

**Supplementary Information:**

The online version contains supplementary material available at 10.1186/s12940-021-00825-9.

## Introduction

Air pollution is one of the main concerns for the health of European citizens (EEA [18]), and the body of evidence on both short- and long-term health effects of air pollution is growing [10, 29, 34, 36, 38]. Following the new findings, the World Health Organization (WHO) has recently updated the Air Quality Guidelines (AQGs) to provide guidance on the health risks of air pollution to the public, especially to policy and other decision makers [44]. In the WHO document, new recommendations on AQ levels are formulated, together with more restrictive interim targets. For instance, the annual mean limit value for NO_2_ has been reduced to a quarter passing from 40 to 10 μg/m^3^.

Regarding this topic, a special concern is given to the population living in cities that suffers from problems related to inadequate housing and transport, waste management, and heat islands. Urban traffic-related emissions are usually more intense [5, 26] and air quality very often does not comply with normative limit values and very rarely with AQGs. As a consequence, European cities are currently striving to accomplish EU air pollution regulation and, even when they do it, are exposed to levels deemed harmful to human health from WHO. In a recent follow-up survey on air quality management, ten European cities reported communication and citizen engagement issues, as well as the need of proving the effectiveness of policies in terms of reduction of concentrations, among their main challenges [42]. Citizen science has already proved to have great potentialities that need to be taken in consideration by institutions (EEA [19]); it has the opportunity to redefine the relations between environmental epidemiology experts and the lay public, and transform the local production of knowledge into a more inclusive and sustainable process [21]. Besides, the interconnection between air pollution and climate change is well known, with the first highly influenced by meteorological variability, as well as by local characteristics (e.g., urban setting), and by the non-linear relationship between emissions and concentrations. For these reasons, identifying effective mitigation measures is challenging [24]. Italian cities have been in the spotlight because of their poor air quality [30]. Given this picture, engaging citizens, and stressing the significance of traffic restriction policies in terms of better air quality and improved health assume an even greater value for Italy.

In 2020, during the SARS COV2 pandemic, it has been hypothesized that chronic exposure to airborne particulate matter (PM_10_, PM_2.5_) and gaseous pollutants such as ozone and nitrogen dioxide (NO_2_) increases the risk of serious health effects such as atherosclerosis, diabetes, and other comorbid conditions, which have been associated with a higher mortality in COVID-19 infected patients [46]. This knowledge has led many researchers to hypothesize a link between air pollution and worsening of the symptoms and prognosis of COVID-19 [11, 13, 32, 35, 41].

During the first phase of the pandemic, several countries implemented strict containment measures, such as the implementation of national lockdowns. The effect of such exceptional interventions in curbing COVID-19 epidemic still needs to be fully evaluated. However, a rapid review suggests a positive effect of the containment measures on the spread of SARS-COV-2 virus, with a major effect in countries where lockdown started earlier and was more restrictive [8].

Among European countries, Italy has been one of the most affected by the COVID-19 pandemic, with more than 4,200,000 infected and over 126,600 deaths by June 2021, and the first to implement strict containment measures, such as the implementation of national lockdown. From March 10^th^ to May 18^th^, 2020, the whole Italian territory experienced a period of severe lockdown to counteract the spreading of the SARS-COV-2 virus. Restriction to individual mobility and the closure of non-essential businesses made traffic flows dramatically fallen (https://www.tomtom.com/covid-19/country/italy/), with the congestion index for Milan that decreased up to 70% [3]. The lockdown measures can be seen as an unintended but effective experiment to assess the impact of traffic restriction policies on air pollution [25, 31, 43].

In this contribution, we estimate the impact of NO_2_ concentrations on mortality at a neighbourhood scale for the two most populated cities of Italy by comparing the reference 2020 scenario with lockdown with an estimated 2020 scenario without lockdown. To do that, a machine learning approach was firstly used to estimate the impact of lockdown on NO_2_ concentrations and then to study the spatial distribution of NO_2_ for two different scenarios: the reference Scenario 1 with lockdown and the estimated Scenario 2 without lockdown. The analysis was performed by considering data routinely collected (AQN) together with a dataset deriving from a large citizen science monitoring project (“NO_2_, No grazie!”), that took place in Milan and Rome between February and March 2020, just before the Italian lockdown period.

## Material and methods

### Study design and domain

**Fig. 1:**
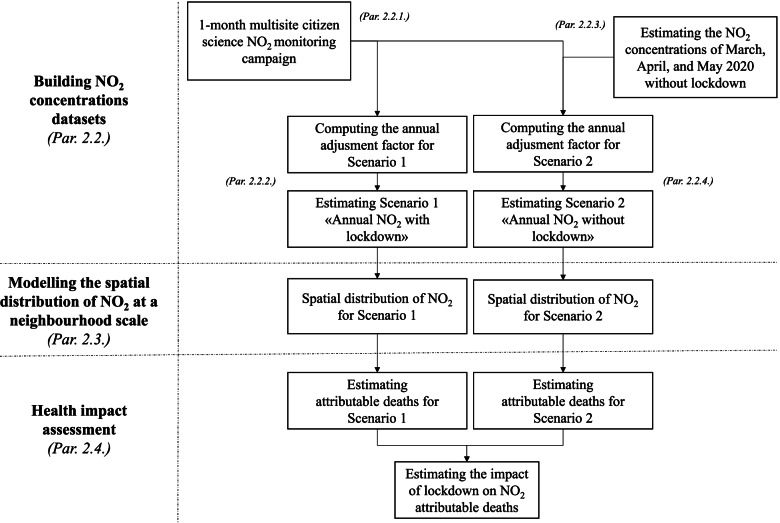
Flowchart of analysis

The design of the project is represented in Fig. 1. Briefly, we used NO_2_ concentration data deriving from a large 1-month multisite citizen science monitoring campaign (par. 2.2.1) that took place just before the beginning of the national lockdown. Subsequently, for each 1-month NO_2_ we built two different datasets estimating the 2020 annual mean with and without the effect of lockdown. These were named respectively Scenario 1 (par. 2.2.2) and Scenario 2 (par. 2.2.3 and 2.2.4) and were built separately for the two cities by applying different annual adjustment factors. The two datasets were then used to model the spatial distribution of NO_2_ at a neighbourhood scale (par 2.3). On this basis, a health impact assessment was finally performed to derive the avoided attributable deaths due to the lower NO_2_ concentrations related to the lockdown. The procedure is presented in detail in the following paragraphs.

The study domain is represented by Milan and Rome, the two most populated Italian metropolitan cities. Milan is located in the Po Valley, in the North of Italy, one of the most polluted areas in Europe (EEA, 18), with a total area of 182 km^2^ and over 1,405,000 inhabitants (2020). Rome is in central Italy, has a total area of 1,287 km^2^ with a population of over 2,808,000 inhabitants (2020) and is interested by high levels of traffic congestion [40].

### Building NO_2_ concentrations datasets

#### The 1-month multisite citizen science monitoring campaign

NO_2_ concentrations were measured by means of passive samplers (diffusion tubes, by Gradko International Ltd) in the frame of the “NO_2_, No grazie” citizen science project coordinated by the Non-Government Organization (NGO) “Cittadini per l'aria” (www.cittadiniperlaria.org).

The monitoring campaign lasted approximately 28 days from the 8^th^ of February 2020 to the 7^th^ of March 2020, just before the implementation of the national lockdown, and involved about 2000 citizens in Rome, Milan and Naples. Citizens were asked to buy and place a diffusion tube in a location of their choice following some guidelines such as putting the sampler in an open environment at about 3 m height. Participants were then asked to fill a specific paper form or to use an application for smartphone [1] to collect information on the monitoring site (e.g., address, coordinates, id number, height etc.).

The accuracy and precision of the samplers were assessed by placing three/four NO_2_ passive samplers in the close proximity (less than 5 m) of the Environmental Agencies Air Quality Network (AQN) stations of the two cities (AEA 2008). The results of this exercise are reported in Additional file 1: A.1.

After the end of the monitoring campaign, samplers were centrally collected and sent to Gradko’s laboratory for quantification analysis (i.e., UV visible spectrometry). We excluded samplers placed outside the administrative border of Milan and Rome, those located in indoor environment or at a height of less than three meters. We also excluded samplers with NO_2_ measured concentrations outliers, identified as follow:

$$x<25p-3*IQR or x>75p+3*IQR$$

with p representing the percentile (25^th^ or 75^th^) and IQR being the Interquartile Range (i.e., the difference between 75 and 25^th^ percentiles) of the entire pool of measures. This represents a common statistical method to identify strong outliers that might be misleading (e.g., instrumental errors). The final number of included samplers were 279 and 287 for Milan and Rome, respectively.

For each sampler, the final 1-month mean NO_2_ was finally obtained by applying an accuracy factor equal to 0.92. The latter was obtained as follow: firstly, for each AQN station, we computed the ratio between the concentration measured by the appositely co-located passive samplers and the station itself; then, we calculated the average of the ratios.

#### Estimating annual concentrations according to Scenario 1 (annual NO_2_ with lockdown)

For each 1-month NO_2_ concentration measured during the citizen science monitoring campaign, a 2020 annual mean was estimated. For the Scenario 1 each 1-month NO_2_ was divided by a specific annual adjustment factor. The latter was calculated according to the following process: 1) The AQN stations of the two cities were identified and data were acquired with a daily time resolution for the entire 2020; 2) for each AQN station, a ratio was calculated by dividing the mean NO_2_ measured from the 8^th^ of February to 7^th^ of March 2020, i.e., the same monitoring period of the 1-month multisite citizen science monitoring campaign, to the 2020 annual mean NO_2_; 3) an average of the ratio was then computed separately for the two cities representing the two city-specific annual adjustment factors; 4) finally, the factors were applied to each 1-month NO_2_ concentration measured during the citizen science monitoring campaign to estimate the mean value for each citizen science monitoring site.

To assess the uncertainty related to the annual estimates, due to the spatial distribution of the AQN stations, the minimum and the maximum ratios for each city were also considered as annual adjustment factors.

#### Estimating the NO_2_ concentrations of March, April, and May 2020 without lockdown

The first step to compute the city-specific annual adjustment factors to build Scenario 2 was to estimate the NO_2_ concentrations of March, April, and May 2020 for each AQN station of the two cities.

We used a machine learning approach, the Random Forest (RF) modelling. The RF is an ensemble method because it aggregates the results of different models: given a training set T, it constructs classifiers h(**x**, T_k_) from each boostrap training sets T_k_ and, then, aggregates the single models for obtaining the bagged prediction. For each (y,**x**) in the training set it aggregates the predictions only over those classifiers for which T_k_ does not contain (y,**x**) [7]. This is the out-of-bag (OOB) classifiers: OOB are data not included in the bootstrap samples at each iteration of the forest and they are considered as a validation set.

The RF adds an element of randomness to the bagging approach, that depends on independent trees constructed using bootstrap samples of the original data set [33]. So, it works by building regression trees, where each node is split based on the best split among some predictors randomly chosen. Finally, the output from each single tree is averaged to obtain a final prediction: the RF model does not overfit because it joins independent trees [7]. The parameters to choose for the model are the number of predictors for each tree, the number of trees, and the target node size [45]. For this study, we used monthly mean NO_2_ measured during the previous 4 years (2016–2019) by the AQN stations as response variables, and dew point (as a proxy of humidity), temperature, pressure at sea level, cumulative rain, wind direction and wind speed, the month, and the ID of each AQN station as predictors. The meteorological variables were chosen according to their association with NO_2_ concentrations and were obtained from the climate Copernicus database (https://climate.copernicus.eu/). The RF models were constructed with the aim to optimize the R^2^ (percent of explained variability) calculated from the out-of-bag (OOB) samples and the root mean squared error (RMSE, μg/m^3^). We also calculated R^2^ (percent of explained variability) from linear relationship between predicted and observed values, root mean square prediction error (RMSPE, μg/m^3^), intercept (μg/m^3^) and slope (μg/m^3^) as fitting statistics.

These models were then used to predict NO_2_ concentrations of March, April, and May 2020 for each station. According to this approach, predicted values represent the expected NO_2_ concentrations for March, April, and May 2020 if the lockdown had not been implemented. A similar approach was recently proposed to assess the impact of lockdown on air quality at a regional scale in Lombardy [23].

The RF is a non-parametric machine learning model: there is not a direct method to quantify the prediction error [14]. For counting the possible range of variation of the unobserved continuous univariate response, we considered the OOB prediction interval as proposed by Zhang et al. [47].

Once predictions for the unobserved places were obtained, we considered the OOB prediction interval for the estimated concentrations, based on the distribution of the RF prediction error $D=Y-\hat{Y}$. To obtain the information about the prediction error distribution, for each iteration *i* = *1, …, n* the error from the subtree considered in the i^th^ iteration was calculated. The OOB prediction $\hat{Y}_{i}$ [7] can be expressed as *w’*_*i*_*Y*, where *w’*_*i*_ rapresents a vector of nonnegative weights that sum to 1. Because *(X1, Y1), …, (Xn, Yn), (X, Y)* are independent and identically distributed, the OOB prediction errors D_1_, …, D_n_ are identically distributed and have approximately the same distribution as D.

We considered prediction interval for Y with approximate coverage probability 1-α, where α = 0.05:

$$[\widehat{\mathrm{Y}}+{D}_{\left[n,\frac{\alpha }{2}\right]}; \widehat{\mathrm{Y}}+{D}_{\left[n,1-\frac{\alpha }{2}\right]}]$$

#### Estimating annual concentrations according to Scenario 2 (annual mean NO_2_ without lockdown)

For each 1-month NO_2_ concentration measured during the citizen science monitoring campaign, a new 2020 annual mean was estimated. As for Scenario 1, for Scenario 2 we divided the 1-month NO_2_ by a specific annual adjustment factor. However, this time such factor was calculated as follow: 1) The AQN stations of the two cities were identified and data were acquired with a daily time resolution for the entire 2020; 2) for each AQN station, a new 2020 annual mean NO_2_ was computed after replacing monthly measured NO_2_ of March, April, and May with those estimated according to par 2.2.3.; 3) for each AQN station, a ratio was calculated by dividing the mean NO_2_ measured from the 8^th^ of February to 7^th^ of March 2020, i.e., the same monitoring period of the 1-month citizen science monitoring campaign, to the new 2020 annual NO_2_; 4) an average of the ratio was then computed separately for the two cities; 5) finally, the factors were applied to each 1-month NO_2_ concentration measured during the citizen science monitoring campaign to estimate a new 2020 mean value, this time without the effect of the lockdown, for each citizen science monitoring site.

As for Scenario 1 (par 2.2.2), uncertainty was assessed by considering as annual adjustment factors the minimum and the maximum ratios. Besides, for each city we further integrate the uncertainty linked to the RF model (par. 2.2.3) by considering the OOB prediction intervals (par 2.2.3.).

### Modelling the spatial distribution of NO_2_ at a neighbourhood scale

The NO_2_ spatial distribution at a neighbourhood scale was estimated by using Land Use Random Forest (LURF) models. These are machine learning algorithms that account air pollutant annual concentration as response variable and a bunch of spatial variables as predictors, to characterize each element of the sample and explain the measured response variable variability. The estimated LURF model is then used to predict pollutant concentrations in places with no-observed measures. This approach is increasingly used in recent studies because it manages to capture complex relationships, even non-linear ones, between predictors and response variables [4].

We built several spatial variables at different circular buffers (20, 50, 100, 300, 500, 1000 m) around each georeferenced NO_2_ passive sampler and, also, around centroids of the grid cells with the aim to use them as predictors for the LURF modelling (Table 1). The residential population was collected by National Census October 2011 from Italian Statistical Institute (ISTAT) for Rome and updated to 2019 by the Municipality for Milan. Land cover predictors were calculated from the Corine Land Cover database (2012) and the DUSAF database of Lombardy Region (2018) as the percentage of each buffer covered by continuous/discontinuous urban fabric, industrial units, green urban areas and forest and semi natural areas; furthermore, the distances from the nearest airport and port were calculated. The length of the roads in meters and traffic loads (calculated as length*vehicle density) by types of roads (highway, major, secondary, local) and by types of vehicles and the distance from the highway were collected from TeleAtlas road network (2012) and the transport municipal Agencies (2017). The number of traffic lights was calculated as a proxy of road crossings for each buffer from OpenStreetMaps data (2019). Finally, mean elevation, imperviousness surface areas and light at night indicators were collected from Copernicus Land Monitoring Service (CLMS) (2012) and Visible Infrared Imaging Radiometer Suite (VIIRS) Day/Night Band (DNB) (2015); only for Milan, sky view factor was collected from an elaboration done from DEM/DSM database by Lombardy Region (2017). The same fitting statistics described in 2.2.3 were calculated for the LURF models trained to predict the NO_2_ concentrations.

**Table 1:** Description of the spatial variables

Variable	Description	Source
**Study domain**	Milan: 73,415 50 × 50 km^2^ grid cells	-
	Rome: 21,332 250 × 250 km^2^ grid cells	-
**Population**	Milan: Resident population in 2019 by census block	Municipality of Milan
	Rome: Resident population from census October 2011	ISTAT
**Land cover**	Land cover characteristics	EEA/Lombardy Region
**Roads**	Road lengths (meters within the cell) and traffic density by road type: highway, major, secondary, and local. Distance between centroid and the major closest road	TeleAtlas TomTom network/Amat
**Traffic signals**	Spatial distribution of traffic lights	OSM
**Imperviousness surface areas**	An indicator of the spatial distribution of artificial areas	EEA—CLMS
**Elevation**	European Digital Elevation Model EU-DEM	EEA—CLMS
**Light at night**	Satellite-based night-time imagery	VIIRS—DNB
**Sky view factor**	Elaboration from Regional Digital Elevation Model	Lombardy Region

Finally, the models were used to predict a NO_2_ value for each centroid of the two grids specifically developed for both cities. In fact, due to the different spatial areas, we divided the Milan spatial domain into 50 × 50 m^2^ grid cells and the Rome spatial domain into 250 × 250 m^2^ grid cells. To consider the uncertainty linked with the LURF models we considered OOB prediction interval for NO_2_ concentrations estimated on the two grids. The procedure was the one already described in par 2.2.3.

### Health impact assessment

Population exposure was obtained on the model grid, where the concentration of pollutants is made available, by aggregating the inhabitants detailed by census blocks.

The population and mortality data for the year 2019 were provided by Italian Institute of Statistics (ISTAT). For Rome, population and mortality data were available at census block. For Milan population data were available at census block, while mortality data at a city borough scale (an aggregation of 82 census blocks on average). In this last case, we redistributed data to the census block level by weighting according to their population size.

For both cities each census block was then intersected with the NO_2_ grid cells: the NO_2_ annual concentration was finally calculated as the mean value from the cells intersecting the census block.

The concentration–response function for all cause-mortality was taken from the "Mortality and Morbidity Effects of Long-Term Exposure to Low-Level PM2.5, BC, NO2, and O3: An Analysis of European Cohorts in the ELAPSE Project” report by the Health Effect Institute of Boston [27]: the relative risk (RR) attributable to NO_2_ exposure was 1.04 (95% CI 1.02—1.07), i.e., mortality increase by 4% every 10 µg / m^3^. Following the 2021 WHO AQGs, a threshold of 10 µg/m^3^ was applied.

The impact of air pollution on the health of the resident population was assessed in terms of NO_2_ attributable deaths, applying the following formula for each census block:

$$attributable\kern0.5em deaths= AF\ast D$$

where

$$AF= \frac{\mathit{exp}\left(\frac{\mathit{log}\left(RR\right)}{10}*counterfactual\right)-1}{exp\left(\frac{\mathit{log}\left(RR\right)}{10}*counterfactual\right)}$$

represents the proportion of mortality attributable to air pollution levels above the counterfactual threshold of 10 µg / m^3^.

In the main analysis we considered:

1. average annual adjustment factors as described in par 2.2.2. and in par 2.2.4.
2. point estimates of the RF models as described in par 2.2.3
3. point estimates of LURF models as described in par 2.3

Moreover, in the attempt to estimate possible variation ranges of NO_2_ attributable deaths, according to the uncertainty related to the different stages of the analysis, we also considered:

1. Minimum and maximum rate as annual adjustment factors as described in par 2.2.2 for Scenario 1 and in par 2.2.4. for Scenario 2;
2. OOB prediction interval computed for monthly estimated NO_2_ of March, April and May as described in par 2.2.3. for Scenario 2;
3. OOB prediction interval computed for annual NO_2_ concentrations estimated on the grids as described in par 2.3 for Scenario 1 and Scenario 2.

The number of attributable cases was also calculated by sub-metropolitan areas (districts) both in Milan and Rome.

All statistical analyses were performed using R software (4.0.3): the *ranger* package for implementation of the Land Use Random Forest models [45] and the *rfinterval* package for the OOB prediction interval [47].

## Results

### Summary statistics of the 1-month multisite citizen science monitoring campaign, Scenario 1, and Scenario 2

Summary statistics of the 1-month measured NO_2_ concentrations as well as annual NO_2_ estimates for Scenario 1 (with lockdown) and Scenario 2 (without lockdown) are reported in Table 2 for Milan and Rome.

**Table 2:** Statistics of the measured citizen science NO_2_ 1-month mean and the estimated annual mean (µg/m^3^) according to Scenario 1 (lockdown) and Scenario 2 (without lockdown) for Milan and Rome

City	No. passive samplers	Data	Mean	SD	Percentiles				
					**5**	**25**	**50**	**75**	**95**
Milan		Measured	50.1	8.9	38.8	44.4	49.1	54.3	67.0
	279	Scenario 1	33.4	5.9	25.9	29.6	32.7	36.2	44.7
		Scenario 2	38.0	6.7	29.4	33.7	37.2	41.2	50.8
Rome		Measured	39.7	8.9	27.1	33.5	37.9	45.3	57.6
	287	Scenario 1	38.6	9.0	26.3	32.5	36.8	44.0	55.9
		Scenario 2	45.7	10.7	31.1	38.5	43.6	52.0	66.2

Between the 8^th^ of February 2020 to the 7^th^ of March 2020 (i.e., the monitoring period of the citizen science project), we measured higher NO_2_ values for Milan than Rome, while the opposite situation was found after having estimated the annual concentrations for Scenario 1. This trend was expected since the peculiar meteorological and geomorphological condition of the Po valley, in which Milan lies, usually determines high pollution events during winter that are more difficult to observe in Rome. Following this condition, the annual adjustment factors for Scenario 1 that were computed by comparing the mean NO_2_ measured by the AQN stations during the citizen science monitoring campaign and the annual mean, was higher for Milan (mean ± SD, 1.50 ± 0.10) than for Rome (mean ± SD, 1.03 ± 0.07).

As explained in par. 2.2.3., to compute the annual adjustment factors for Scenario 2, the first step has been the estimate of the NO_2_ concentrations of March, April, and May without lockdown. This was done by training a RF model starting from the historical monthly data of the AQN stations of the two cities for the 2016–2019 period. Table 3 shows the fitting statistics of the model. The predicted NO_2_ values for March, April, and May 2020 of each AQN monitoring station showed a mean reduction of 42% and 52% in Milan and Rome (Additional file 1: A.2). While when focusing on the annual mean NO_2_, the estimated reduction due to the lockdown was 12% and 15%, respectively (Additional file 1: A.3 and A.4).

**Table 3:** Fitting statistics of the Random Forest model trained to estimate NO_2_ concentrations measured by the AQN monitoring sites for Scenario 2

	**OOB-R**^**2**^	**RMSE**	**pred-R**^**2**^	**RMSPE**	**Inter**	**slope**
	0.78	6.88	0.96	3.1	5.49	0.88

By evaluating the OOB prediction interval of NO_2_ annual concentrations for each AQN station both for Milan and for Rome, we could observe that these values ​were higher than the observed annual concentrations in 2020 (Scenario 1 with lockdown): for each estimated month, for no AQN station the lower extreme of the interval was lower than the value observed in 2020 (Additional file 1: A.5).

Following this approach, the annual adjustment factors for Scenario 2 that were computed by comparing the mean NO_2_ measured by the AQN stations during the citizen science monitoring campaign and the new estimated annual mean, was higher for Milan (mean ± SD, 1.32 ± 0.09) than for Rome (mean ± SD, 0.87 ± 0.06).

### Land Use Random Forest models

Once building Scenario 1 and Scenario 2 for the two cities, the following step has been the analysis of the spatial distribution of the contaminant. The four Land Use Random Forest models (two scenarios for both Milan and Rome) were able to capture 41–42% of the total NO_2_ variability in out-of-bag samples, with a RMSE of 4.5 μg/m^3^ and 5.1 μg/m^3^ for the two scenarios for Milan and of 6.6 μg/m^3^ and 7.8 μg/m^3^ for the two scenarios for Rome (Table 4).

**Table 4:** Fitting statistics of the LURF models trained to predict NO_2_ concentrations on Milan and Rome according to the different scenarios

		**OOB-R**^**2**^	**RMSE**	**pred-R**^**2**^	**RMSPE**	**Inter**	**slope**
**Milan**	**Scenario 1**	0.42	4.5	0.42	3.4	19.4	0.42
	**Scenario 2**	0.42	5.1	0.42	3.9	21.9	0.42
**Rome**	**Scenario 1**	0.46	6.6	0.46	5.2	22.6	0.42
	**Scenario 2**	0.46	7.8	0.46	6.2	26.7	0.41

**Fig. 2:**
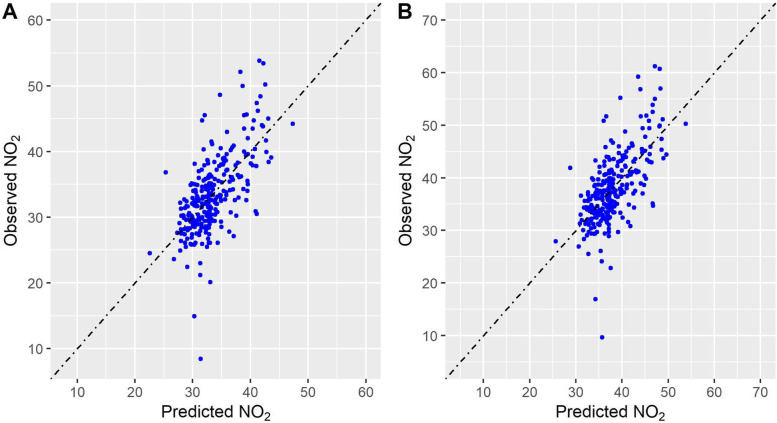
Comparison between observed (y axis) and predicted (x axis) concentrations for Milan – Scenario 1 (**A**) and Scenario 2 (**B**) models

**Fig. 3:**
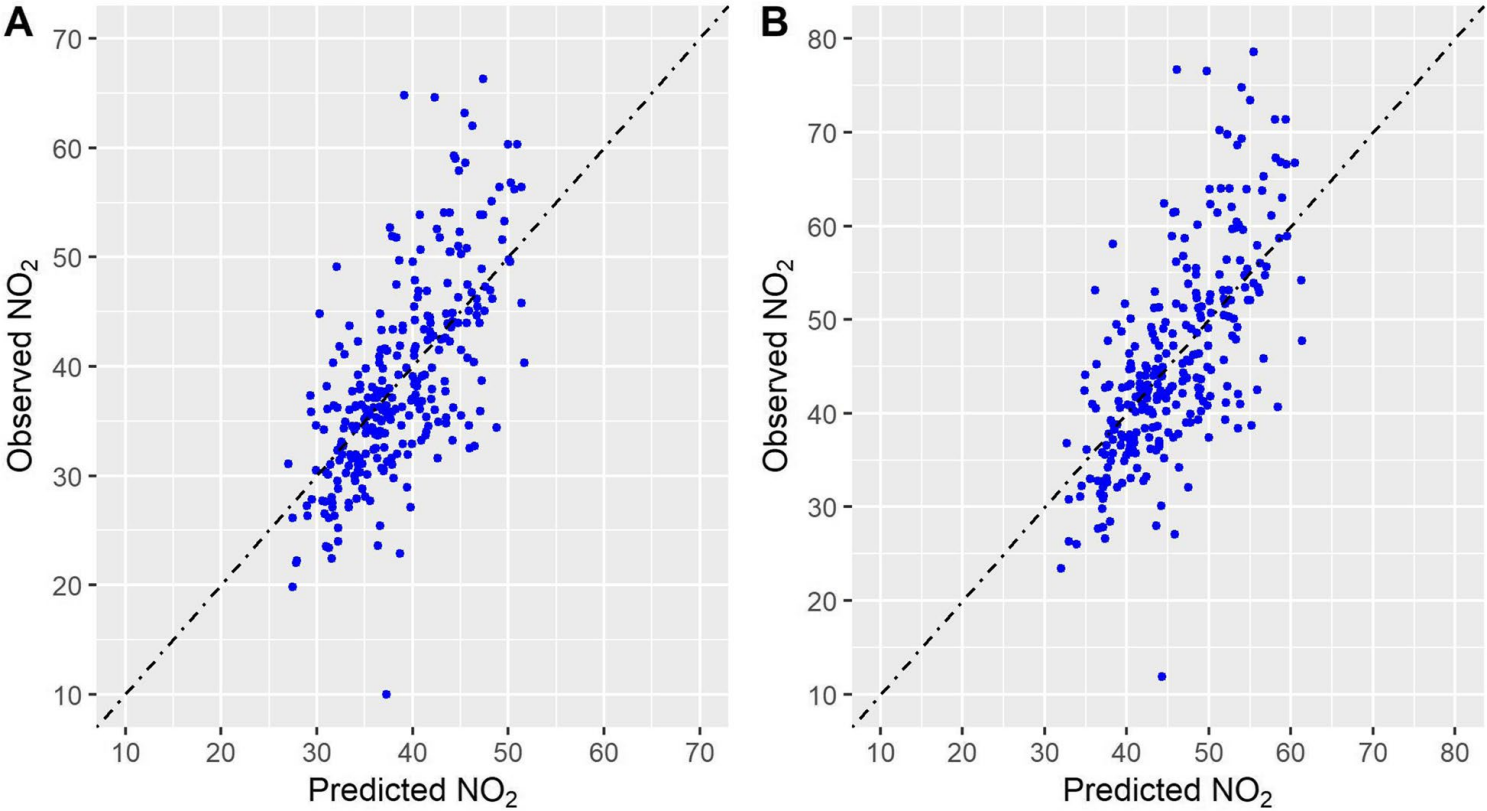
Comparison between observed (y axis) and predicted (x axis) concentrations for Rome – Scenario 1 (**A**) and Scenario 2 (**B**) models

The models slightly overestimated lower values ​​and underestimated higher ones (Fig. 2 and Fig. 3).

For what concern the city of Milan, the mean predicted NO_2_ annual concentration was 29.5 μg/m^3^ (SD 3.7) for Scenario 1 and 33.5 μg/m^3^ (SD 4.2) for Scenario 2 (Table 5A). The population-weighted exposure was 31.7 μg/m^3^ for Scenario 1 and 36.0 μg/m^3^ for Scenario 2. As opposed to Scenario 1, for Scenario 2 we found a significant portion of the population exposed to higher NO_2_ concentrations than the EU limit of 40 μg/m^3^ (5% of the total).

**Table 5:** Statistics of predicted NO_2_ annual mean (µg/m^3^) of the Milan and Rome grids according to the different scenarios

		**No. cells**	**Mean**	**sd**	**Percentiles**				
					**5**	**25**	**50**	**75**	**95**
**Milan**	**Scenario 1**	73,415	29.5	3.7	23.9	26.5	29.4	31.5	36
	**Scenario 2**	73,415	33.5	4.2	27.2	30.4	33.4	35.8	40.9
**Rome**	**Scenario 1**	21,332	29.3	4.0	25.9	26.8	27.4	30.6	38.3
	**Scenario 2**	21,332	34.6	4.7	30.7	31.6	32.3	36.1	45.2

**Fig. 4:**
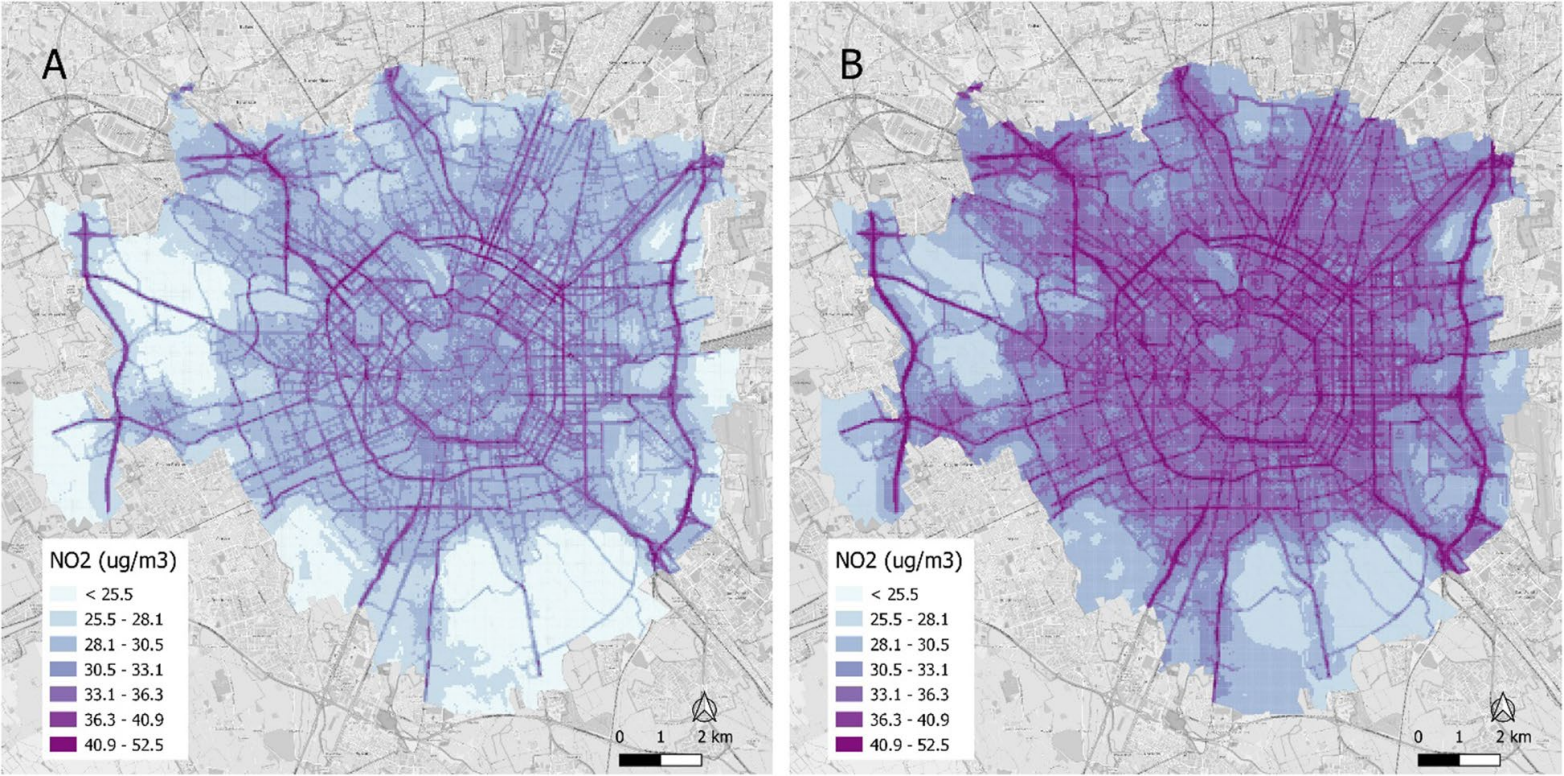
Predicted NO_2_ concentrations for Milan grid according to Scenario 1 (**A**) and Scenario 2 (**B**)

As reported in Additional file 1: A.6, the most important predictors were traffic load for light vehicles and heavy and commercial vehicles and roads length related, above all, to buffers of 20, 50 and 100 m and the distance from the major road. A visual interpretation of the results is given in Fig. 4.

For what concern the city of Rome, the mean predicted NO_2_ annual concentration was 29.3 μg/m^3^ (SD 4.0) for Scenario 1 and 34.6 μg/m^3^ (SD 4.7) for Scenario 2 (Table 5B). The population-weighted exposure was 34.3 μg/m^3^ for Scenario 1 and 40.4 μg/m^3^ for Scenario 2. By comparing the two scenarios, the share of the exposed population to higher NO_2_ concentrations than 40 μg/m^3^ strongly increased passing from 10.5% (Scenario 1) of the total population to 44.8% (Scenario 2).

**Fig. 5:**
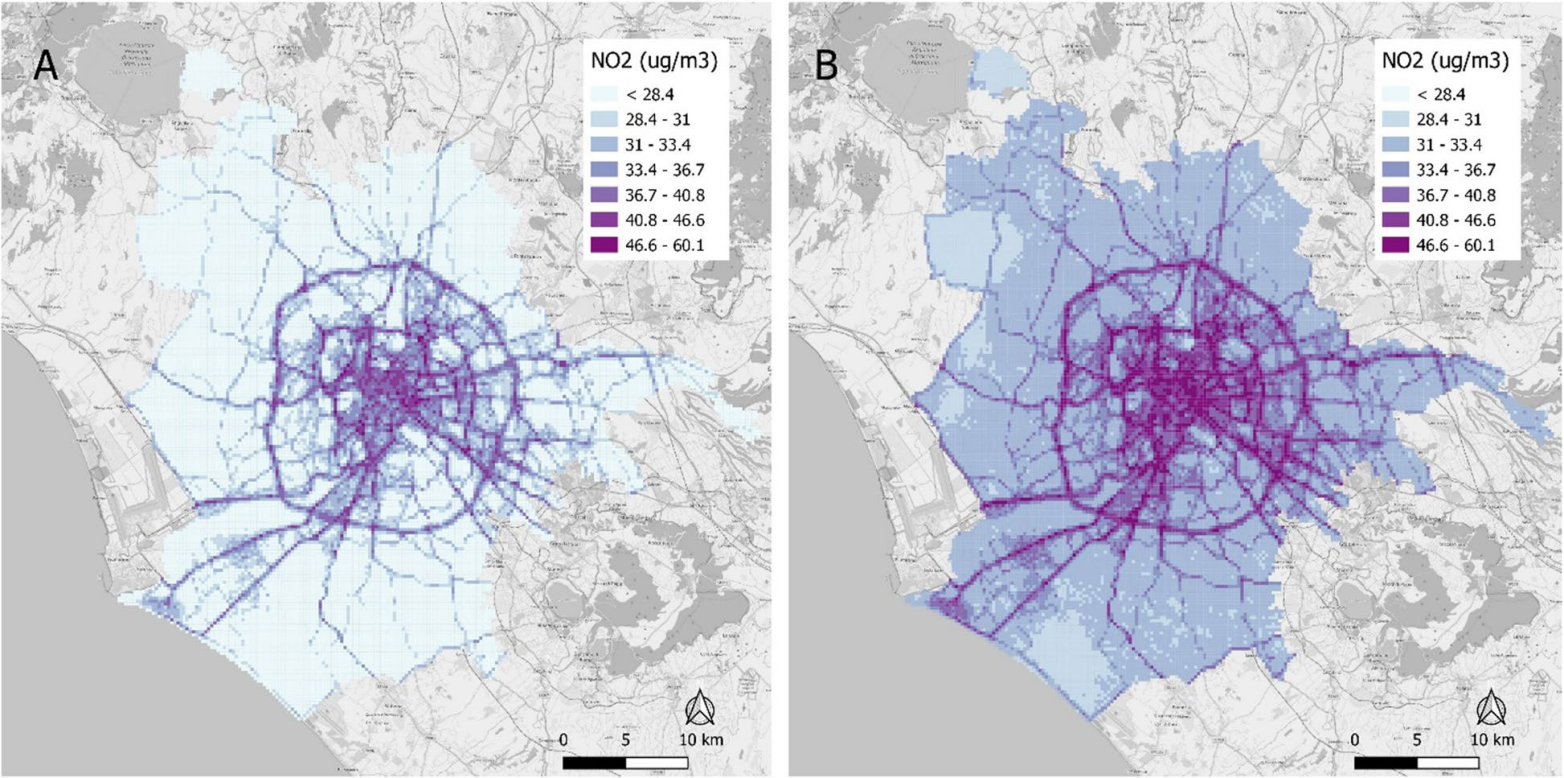
Predicted NO_2_ concentrations for Rome grid according to Scenario 1 (**A**) and Scenario 2 (**B**)

The most important predictors of the LURF models for Rome were, again, traffic load for light vehicles and heavy and commercial vehicles and roads length, but the involved buffers were greater than those found for Milan (Additional file 1: A.7). A visual interpretation of the results is given in Fig. 5.

As it can be seen in Additional file 1: A.8, for neither of the cities the lower extremes obtained by the assessment of the OOB predictions fell below 10 μg/m^3^, the threshold used for the health impact assessment.

### Health impact assessment

For what concern the city of Milan, the number of NO_2_ attributable deaths for a risk threshold of 10 μg/m^3^ went from 1130 (IC95%: 583, 1891) considering Scenario 1 (annual NO_2_ with lockdown) to 1343 (IC95%: 695, 2234) considering Scenario 2 (annual NO_2_ without lockdown) with an increase of 213 deaths (+ 18.9%). The proportion of NO_2_ attributable deaths on the total, changed from 8.2% to 9.7% if considering Scenario 1 and Scenario 2, respectively. Uncertainty intervals of attributable deaths estimates linked with the different stages of the analysis are given in Additional file 1 (Additional file 1: A.9). The uncertainty linked to the estimates of the annual NO_2_ values for the city of Milan (par. 2.2.2) varied as following: 1130 (995–1330) for Scenario 1; 1343 (1184–1330) for Scenario 2. Moreover, for Scenario 2 the range remained constant also after integrating the uncertainty linked to the estimates of the RF model (par. 2.2.3): attributable deaths became 1343 (1207–1504). Finally, the ranges of uncertainty widened if considering the LURF models: attributable deaths were 1130 (653–1603) for Scenario 1 and 1343 (804–1873) for Scenario 2. However, in each case the health advantage represented by Scenario 1 was unchanged: for example, considering the uncertainty linked to the LURF models there was an increase of 270 deaths (+ 17%) from Scenario 1 to Scenario 2.

A similar analysis was conducted for the city of Rome. The number of NO_2_ attributable deaths for a risk threshold of 10 μg/m^3^ went from 2541 (IC95%: 1315, 4230) considering Scenario 1 (annual NO_2_ with lockdown) to 3145 (IC95%: 1637, 5192) considering Scenario 2 (annual NO_2_ without lockdown) with an increase of 604 deaths (+ 23.8%). The proportion of NO_2_ attributable deaths on the total changed from 9.2% to 11.4% if considering Scenario 1 and Scenario 2, respectively. As for Milan, also for Rome Table 9 reports the uncertainty intervals of attributable deaths estimates linked with the different stages of the analysis. The uncertainty linked to the estimates of the annual NO_2_ values for the city of Rome (par. 2.2.2) varied as following: 2541 (2199–2975) for Scenario 1; 3145 (2733–3590) for Scenario 2. Moreover, for Scenario 2 the range remained constant also after integrating the uncertainty linked to the estimates of the RF model (par. 2.2.3): attributable deaths became 3145 (2788–3544). Finally, the ranges of uncertainty widened if considering the LURF models: attributable deaths were 2541 (1167–3855) for Scenario 1 and 3145 (1543–4683) for Scenario 2. However, in each case the health advantage represented by Scenario 1 was unchanged: for example, considering the uncertainty linked to the LURF models there was an increase of 828 deaths (+ 21%) from Scenario 1 to Scenario 2.

As for the HIA at districts level, Additional file 1: A.10 shows the location of the districts in the two cities, while general information and descriptive statistics of NO_2_ concentrations, attributable deaths, and mortality rates are given for Scenario 1 and Scenario 2 in Additional file 1: A.11 for Milan and Additional file 1: A.12 for Rome. Results are different for the two cities: the relative increment of attributable mortality rates passed from 18.3% to 19.1% in Milan (9 districts) and from 22.6% to 25.5% in Rome (15 districts).

## Discussion

In this study, the impact of the national lockdown measures implemented because of the COVID19 pandemic between March and May 2020 on NO_2_ concentrations and attributable deaths were assessed for Milan and Rome in Italy. The analysis was conducted by using both AQN stations and data collected in a large citizen science monitoring project called “NO_2_, No grazie!”. A machine learning approach was used to model NO_2_ concentrations at a neighbourhood scale, while attributable deaths were estimated at the finest possible spatial scale (census block) by following the most recent findings in the field. We found an important, albeit short-term, reduction in concentrations of NO_2_, a pollutant highly related to traffic, with a consequent population-weighted exposure reduction of 15.1% in Milan and 15.3% in Rome on an annual basis. The resulting avoided estimated attributable deaths were 213 and 604 for Milan and Rome, respectively. Besides, the impact of the lockdown on attributable deaths showed differences inside the two urban areas. Finally, even considering the sources of uncertainty linked to the different models that were used in this study, Scenario 2 showed a greater amount of NO_2_ attributable deaths than Scenario 1.

Meteorological conditions and non-linear relationship between emissions and concentrations make the evaluation of the effect of local interventions more challenging, and Random Forest models have already proved to be an effective approach to address this issue [24]. However, to the best of our knowledge, only a few studies have used such an approach to assess the impact of the lockdown measures on air quality [11, 23, 37]. In Italy, Granella et al. [23] used a machine-learning based approach to estimate the impact of lockdown on air quality in the whole Lombardy, the Italian region where Milan is located. They used the prediction error of the estimates compared to the measurements of different air pollutants computed for all the AQN monitoring sites. For NO_2_, they estimated a reduction of about 33% (absolute value 10 μg/m^3^) for the whole Lombardy region. This reduction is comparable to our result (42%, 20.5 μg/m^3^), especially if considering that our analysis was focused only on urban environment where traffic source is the most impactful [5].

Some studies evaluated the impact of lockdown measures on air pollution also for the city of Rome [6, 39]. Sicard et al. [39] analysed short-term changes at 36 urban stations in Nice (France), Rome and Turin (Italy), Valencia (Spain) and Wuhan (China) from the 1^st^ of January 2017 until the 18^th^ of April 2020, with the aim of comparing 2020 with the previous 3 years. In detail, the 15 analysed stations for Rome showed a decrease in NO_2_ concentrations equals to 50.3% on weekdays and 42.2% on weekends; these results are comparable with the 52% reduction observed in our analysis.

Furthermore, Gualtieri et co-workers (2020) presented an analysis to assess the impact of lockdown measures on air quality in some Italian cities, including Milan and Rome, comparing a lockdown scenario (year 2020) and a scenario without lockdown (2019). As in our study, results were obtained for Milan and Rome through a homogeneous methodology. In Milan, where mobility decreased on average by 60%, the decrease of NO_2_ was of 34.8% at urban background (UB) stations and of 24.9% at urban traffic (UT) ones. In Rome, otherwise, an average decrease of 65% was observed in mobility rate with a consequent decrease in NO_2_ concentrations of 46.8% at UT and 41.6% at UB stations.

Considering the entire territory of the two cities, an increase of NO_2_ mean concentrations equal to 13.6% for Milan and 18.1% for Rome were observed shifting from Scenario 1 (annual NO_2_ with lockdown) to Scenario 2 (annual NO_2_ without lockdown). Consequently, also the attributable deaths linked to the NO_2_ concentrations marked a strong increase: + 18.9% and + 23.8%, respectively for Milan and Rome when considering a threshold of 10 μg/m^3^. Besides, it is worth to underline that although the two cities apparently complied with the EU legal annual threshold of 40 μg/m^3^, the estimated attributable deaths were more than 1000 and 2000 deaths considering Scenario 1 (annual NO_2_ with lockdown) for Milan and Rome, respectively. The updated WHO Global Air Quality Guidelines (AQGs) have provided recommendations on air quality threshold levels as well as interim targets for six key air pollutants, including NO_2_. Meeting the interim targets may have a notable benefit for health, especially in areas where exposures far exceed these values. As recently underlined by the Health Effect Institute of Boston [27], given the evidence that health effects occur all the way down to very low concentration levels, future clean air policies must include incentives for progressive lowering of exposures of the entire population, thereby improving health for all. What is needed is a paradigm change from relying solely on fixed limit values, with a shift towards the concept of combining fixed limit values with a continuous reduction of the average exposure. For example, the current European Union (EU) Ambient Air Quality Directive already contains a non-binding average exposure reduction target (EC, 2008). The upcoming 2022 revision of the EU Ambient Air Quality Directive will offer the chance to lead the way and implement binding average exposure reduction goals for air pollutants in combination with lowered fixed limit values [28].

The added values of our study were: this was an example of co-created Citizen Science which implied the full involvement of the NGO and citizens in the planning of the research project with the scientists; modelling spatial distribution of NO_2_ at a neighbourhood scale thanks to a great amount of monitoring data collected by citizens; taking into account the meteorological conditions actually experienced during the lockdown period to estimate a scenario without lockdown (Scenario 2); collecting population data at a very local scale. In this way, it was possible to estimate high spatial resolution NO_2_ concentration maps for Milan and Rome and, through them, to calculate the number of NO_2_ attributable deaths in both scenarios at a neighbourhood spatial scale lowering the bias linked to the use of aggregated data in environmental epidemiology [16].

This study presents some weaknesses as well. Because of the lack of information at a neighbourhood scale, it was not possible to consider other main health effects than attributable deaths. Future studies should focus on this issue since the impact of NO_2_ on urban population health does not limit to mortality but also concerns the morbidity. Besides, in the attempt to estimate annual NO_2_ concentrations from the 1-month data we applied annual adjustment factors for each monitoring site, independently from their location (i.e., urban background or traffic sites). This simplification probably influenced the variability of our original set of monitoring data reducing the capability of LURF models to catch the real spatial contrast inside the study areas possibly reducing the capability to identify heterogeneity in both concentrations and health effects. Further attempts to consider the peculiarities of different classes of monitoring sites should be made to get closer to the real scenario.

Besides, the use of fixed factors to estimate the annual values starting from the 1-month data does not represent the state of the art in the field. For instance, De Craemer et al. [15] used a calibration regression model to extrapolate annual means from co-located diffusion tubes in a multi-week multi-site monitoring campaign. However, since we did not have multi-week data, the fixed factors approach still represented the best method for us.

Finally, our data may suffer from possible citizen science related biases, such as bias in selection of the sample or data collection related issues [20], that may affect the overall interpretation and generalizability of the results. In particular, the geocoding process might have been subjected to inaccuracy, especially for those who did not use the smartphone geocoding application. However, the scientific committee of the project partially tried to overcome these issues by positioning a set of passive samplers in selected areas (i.e. monitoring sites under- and over- exposed to traffic, especially highly dense urban fabric or lower ones, street canyons etc.), aiming at maximize the spatial contrast captured by the original set of monitoring sites, as well as by adopting a selection criteria seeking to ensure outliers and not eligible monitoring sites (i.e. inside courtyards, height major than 3 m, indoor locations etc.).

## Conclusion

The lockdown experience represented an opportunity to decrease road traffic, which is the main cause of NO_2_ air pollution in cities, but in the near future effective environmental policies to reduce traffic-related air pollution and mitigate climate change need to be implemented in our cities. This study shows that this “natural experiment” has been a beneficial opportunity for human health, especially in terms of NO_2_ attributable deaths. Besides, by highlighting the number of deaths due to risk threshold of 10 μg/m^3^, our study calls for more ambitious traffic calming policies in Rome and Milan and a re-evaluation of the annual EU limit values for nitrogen dioxide (NO2) for the protection of human health.

## Authors’ information

Boniardi Luca and Nobile Federica contributed equally to this work.

## Supplementary Information

**Additional file 1.**

## Data Availability

Not applicable.

## Abbreviations

AQN: Air Quality Guidelines
AQN: Air Quality Network
CLMS: Copernicus Land Monitoring Service
DEM: Digital Elevation Model
DNB: Day/Night Band
EU: European Union
HRAPIE: Health risks of air pollution in Europe
IQR: Interquartile Range
ISTAT: Italian Statistical Institute
LURF: Land Use Random Forest
NGO: Non-Governmental Organization
NO2: Nitrogen dioxide
OSM: OpenStreetMaps
OOB: Out-of- bag
PM: Particulate matter
RF: Random Forest
RMSE: Root mean squared error
RMSPE: Root mean square prediction error
SD: Standard deviation
UB: Urban background
UT: Urban traffic
VIIRS: Visible Infrared Imaging Radiometer Suite
WHO: World Health Organization
