# Supplementary material for: A multi-step machine learning approach to assess the impact of COVID-19 lockdown on NO2 attributable deaths in Milan and Rome, Italy
Source: Environ Health. 2022 Jan 16;21:17. doi: 10.1186/s12940-021-00825-9 (PMC8761378; doi:10.1186/s12940-021-00825-9)
Supplement: Supplementary file 1 — Additional file 1. [file 12940_2021_825_MOESM1_ESM.docx]

**Additional file 1**

**A.1** Accuracy and precision of the passive samplers.

| **AQN station** | **ID** |  | **Class** | **City** | **No. passive  samplers** | **Accuracy (Error%)** | **Precision (Sd%)** |
| --- | --- | --- | --- | --- | --- | --- | --- |
| San Giuliano | 606 | ARPA Lombardia* | UT | Milan | 3 | 13.7 | 2.8 |
| Senato | 548 |  | UT | Milan | 3 | 17.0 | 8.6 |
| Liguria | 539 |  | UT | Milan | 3 | -6.9 | 0.5 |
| Magna Grecia | 05 | ARPA Lazio* | UT | Rome | 4 | 8.0 | 5.7 |
| Fermi | 47 |  | UT | Rome | 3 | 9.8 | 22.1 |
|  |  |  |  |  | Mean | 9.5 | 7.9 |

*ARPA Lombardia – https://www.dati.lombardia.it/Ambiente/Stazioni-qualit-dell-aria/ib47-atvt
ARPA Lazio – http://dati.lazio.it/catalog/it/dataset/rete-di-monitoraggio-della-qualita-dell-aria

**A.2** Actual and predicted NO_2_ concentrations for each monitoring AQN station of Milan (A) and of Rome (B).

**A)**


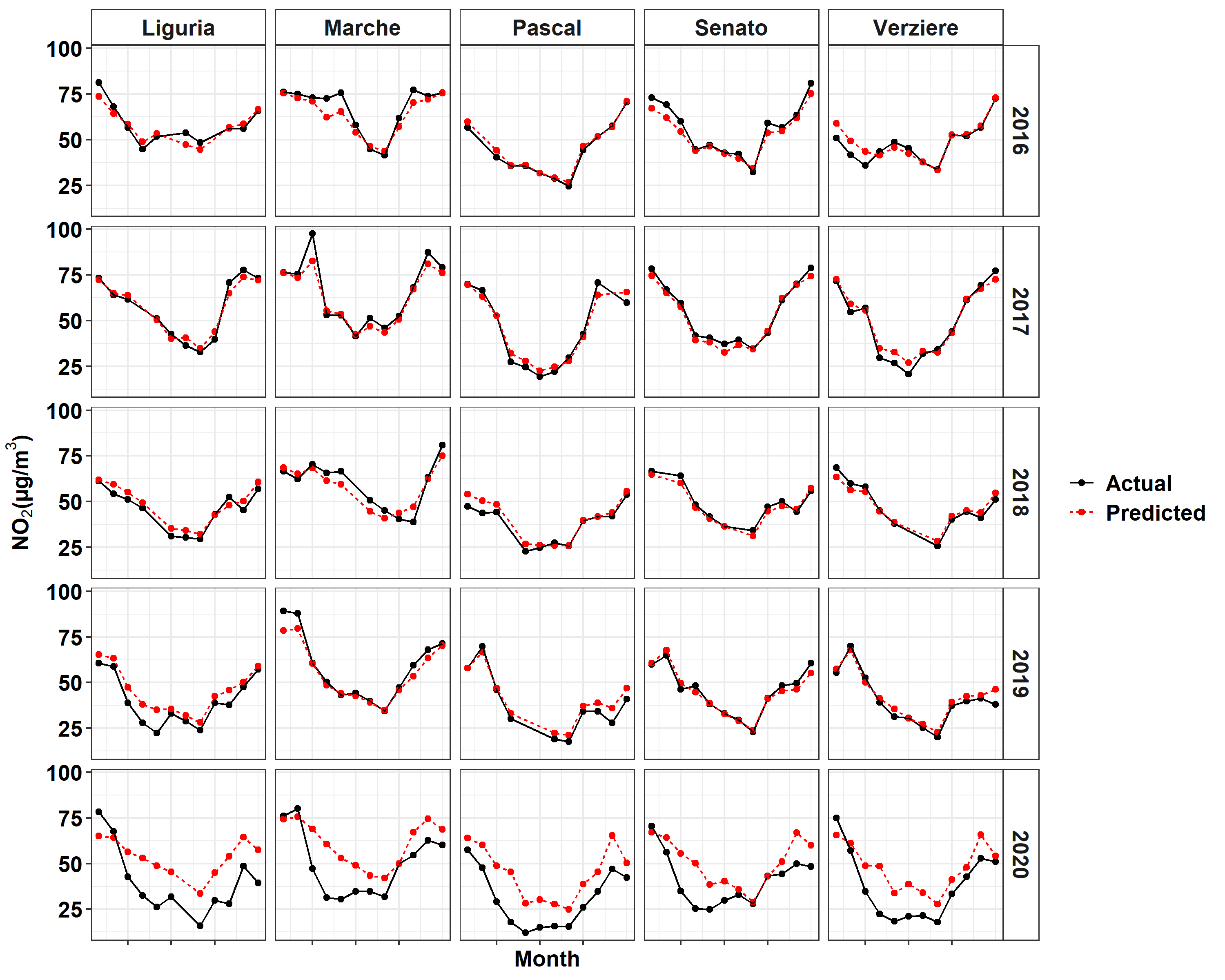


**B)**


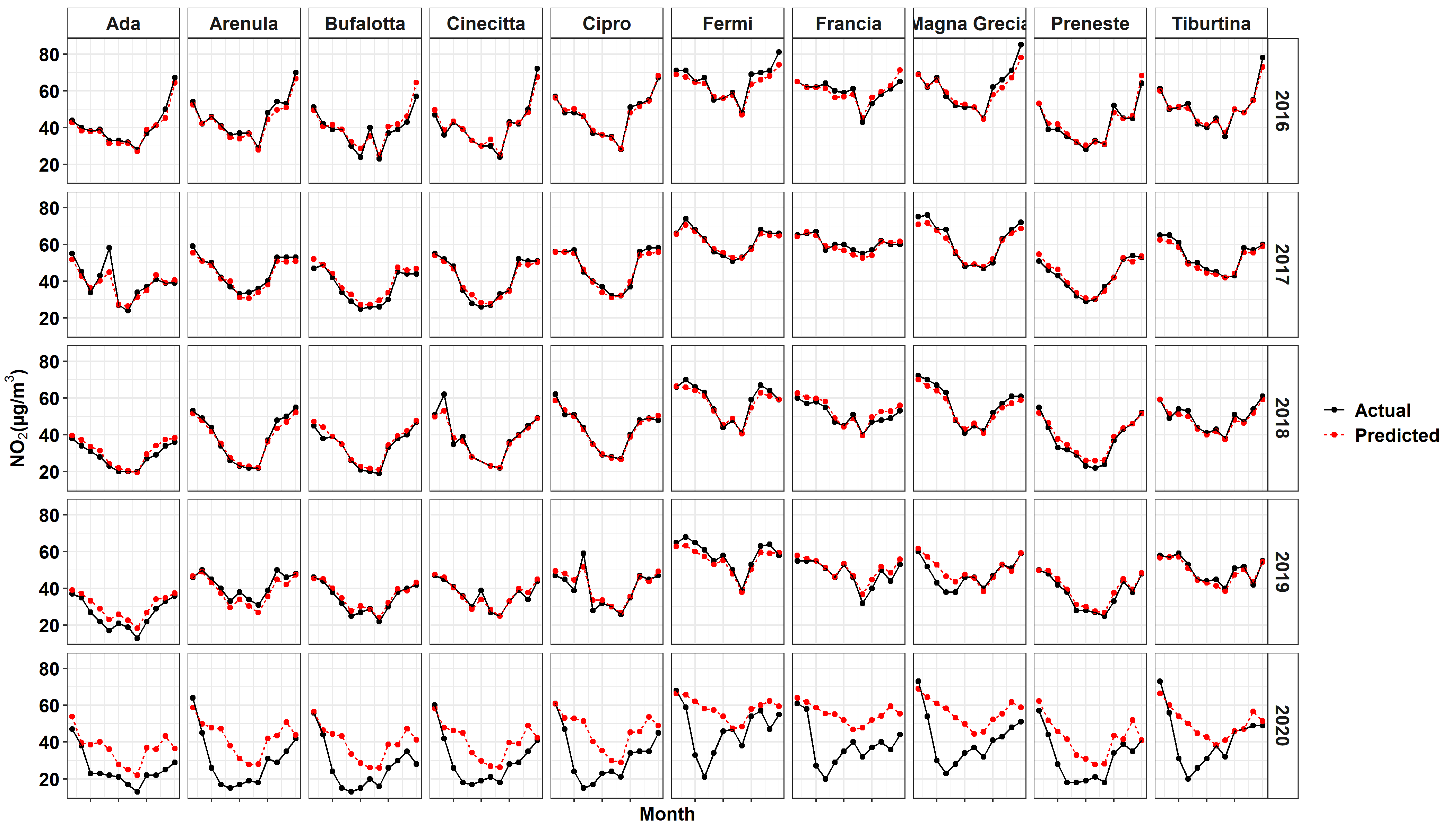


**A.3** Statistics related to the AQN monitoring sites of Milan. A) is the mean NO_2_ measured during the period of the citizen science project; B) is the measured annual mean NO_2_ for 2020; C) is the estimated annual mean for 2020 without the lockdown; A/B is the factor used to estimate the annual mean NO_2_ of the passive samplers for 2020; A/C is the factor used to estimate the annual mean NO_2_ of the passive samplers for 2020 without lockdown.

| **ID** | **AQN site** | **Class** | **NO_2_ (08/02-07/03)** | **NO_2_ 2020** | **NO_2_ no lockdown** | **(B-C)/C** | **A/B** | **A/C** |
| --- | --- | --- | --- | --- | --- | --- | --- | --- |
| 539 | Liguria | Urban traffic | 65.7 | 40.1 | 45.2 | -11% | 1.64 | 1.45 |
| 501 | Marche | Urban traffic | 76.3 | 49.5 | 55.6 | -11% | 1.54 | 1.37 |
| 705 | Pascal | Urban background | 45.4 | 30.1 | 35.3 | -15% | 1.51 | 1.29 |
| 548 | Senato | Urban traffic | 54.1 | 40.7 | 45.6 | -11% | 1.33 | 1.19 |
| 528 | Verziere | Urban traffic | 54.8 | 37.3 | 42 | -11% | 1.47 | 1.31 |
|  |  | mean | 59.3 | 39.5 | 44.7 | -12% | 1.50 | 1.32 |
|  |  | sd | 11.9 | 7 | 6.6 | 2% | 0.10 | 0.09 |

**A.4** Statistics related to the AQN monitoring sites of Rome (inner city). A) is the mean NO_2_ measured during the period of the citizen science project; B) is the measured annual mean NO_2_ for 2020; C) is the estimated annual mean for 2020 without the lockdown; A/B is the factor used to estimate the annual mean NO_2_ of the passive samplers for 2020; A/C is the factor used to estimate the annual mean NO_2_ of the passive samplers for 2020 without lockdown.

| **ID** | **AQN site** | **Class** | **NO_2_ (08/02-07/03)** | **NO_2_ 2020** | **NO_2_ no lockdown** | **(B-C)/C** | **A/B** | **A/C** |
| --- | --- | --- | --- | --- | --- | --- | --- | --- |
| 39 | Ada | Urban background | 28.2 | 25.2 | 29.1 | -13% | 1.12 | 0.97 |
| 56 | Arenula | Urban background | 32.8 | 29.9 | 36.1 | -17% | 1.10 | 0.91 |
| 48 | Bufalotta | Urban background | 30.7 | 27 | 32.6 | -17% | 1.14 | 0.94 |
| 08 | Cinecittà | Urban background | 30.2 | 29.5 | 34.9 | -15% | 1.02 | 0.87 |
| 49 | Cipro | Urban background | 32.1 | 32 | 39.1 | -18% | 1.00 | 0.82 |
| 47 | Fermi | Urban traffic | 42.3 | 46.4 | 54.1 | -14% | 0.91 | 0.78 |
| 03 | Francia | Urban traffic | 39.4 | 37.9 | 46 | -18% | 1.04 | 0.86 |
| 05 | Magna Grecia | Urban traffic | 38.7 | 41.2 | 48.8 | -16% | 0.94 | 0.79 |
| 02 | Preneste | Urban background | 32.4 | 31 | 35.7 | -13% | 1.05 | 0.91 |
| 55 | Tiburtina | Urban traffic | 40.8 | 41.2 | 47.5 | -13% | 0.99 | 0.86 |
|  |  | mean | 34.8 | 34.1 | 40.4 | -15% | 1.03 | 0.87 |
|  |  | sd | 5 | 7.1 | 8.2 | 2% | 0.07 | 0.06 |

**A5.** Observed NO_2_ concentrations (Scenario 1) and estimated NO_2_ concentrations with OOB predict interval (Scenario 2) for March, April and May 2020 for each AQN station of Milan (A) and Rome (B)
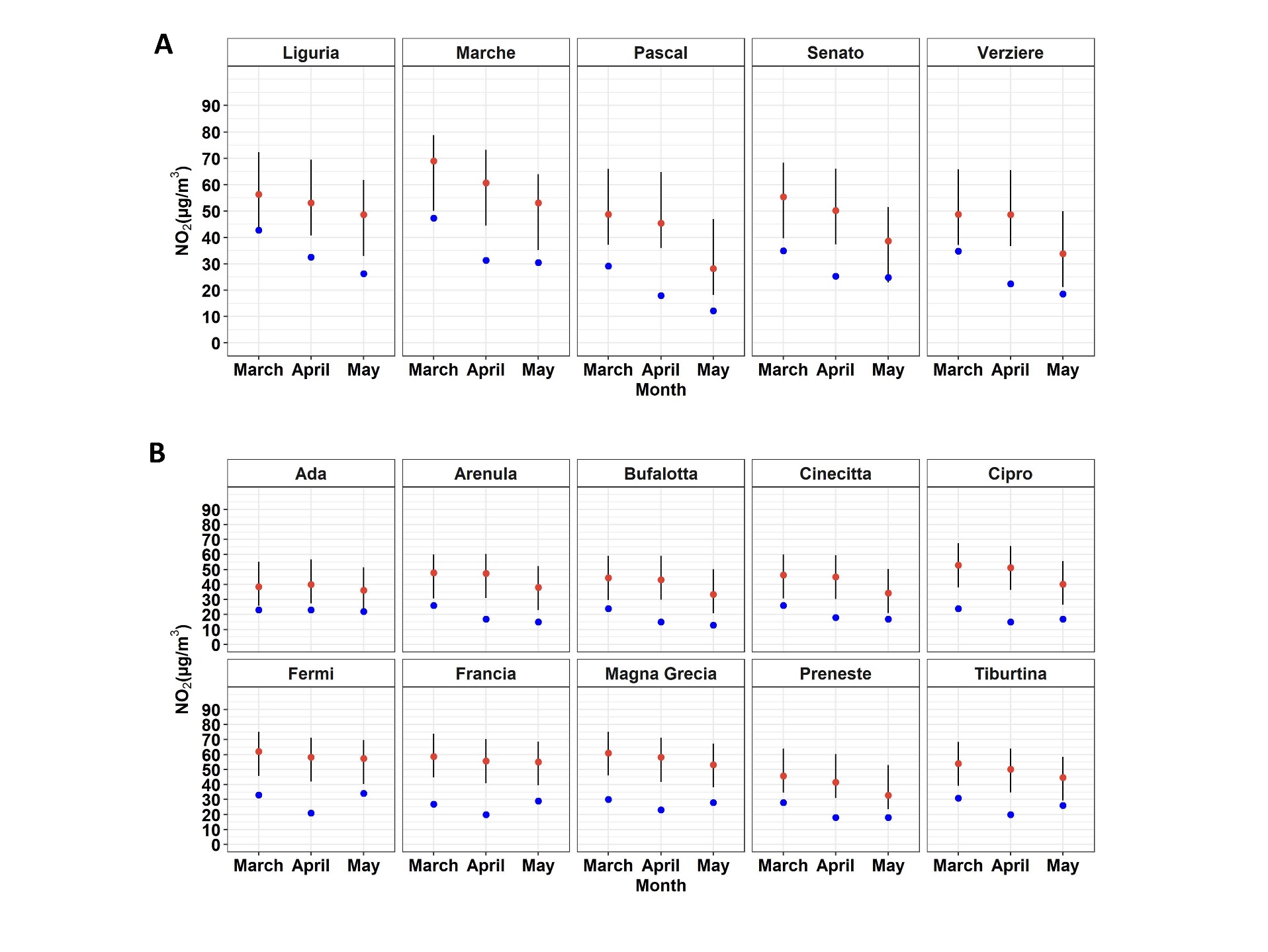


**A.6** Variables relative importance (%) in scenario 1 (A) and scenario 2 (B) LURF models for Milan.


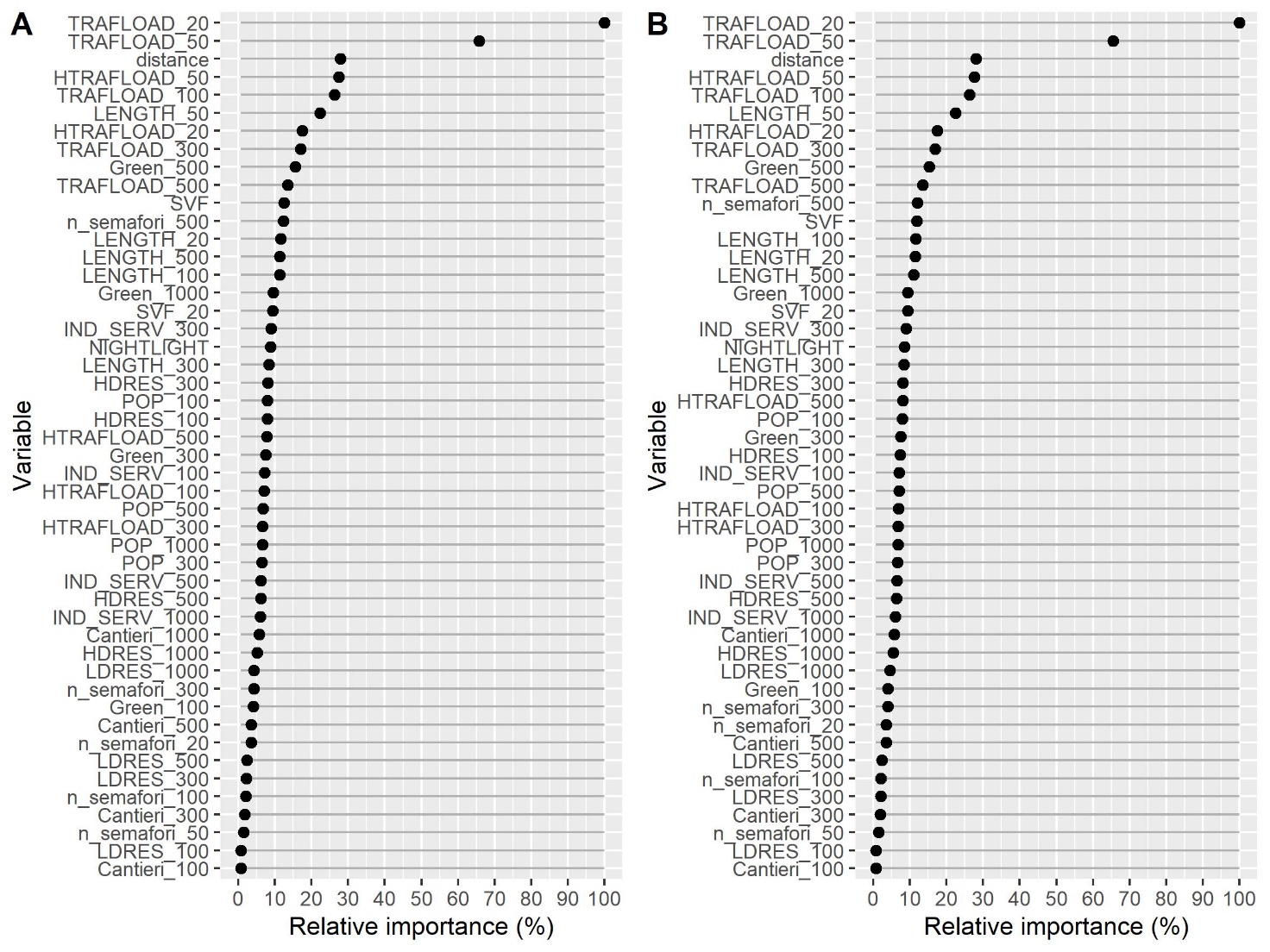


**A.7** Variables relative importance (%) in scenario 1 (A) and scenario 2 (B) LURF models for Rome.


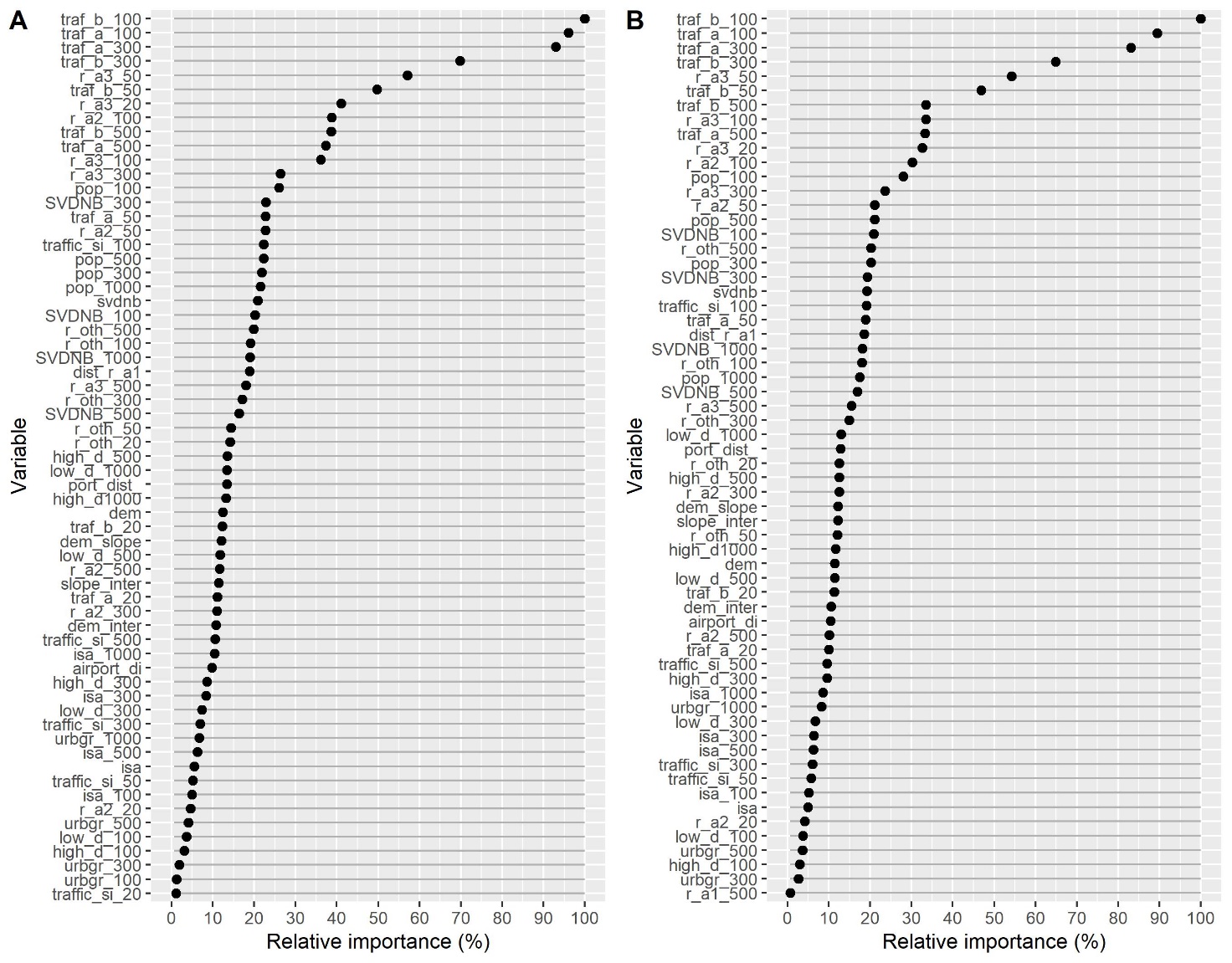


**A.8** NO_2_ concentrations predicted on each grid city for each Scenario and relative OOB prediction intervals.


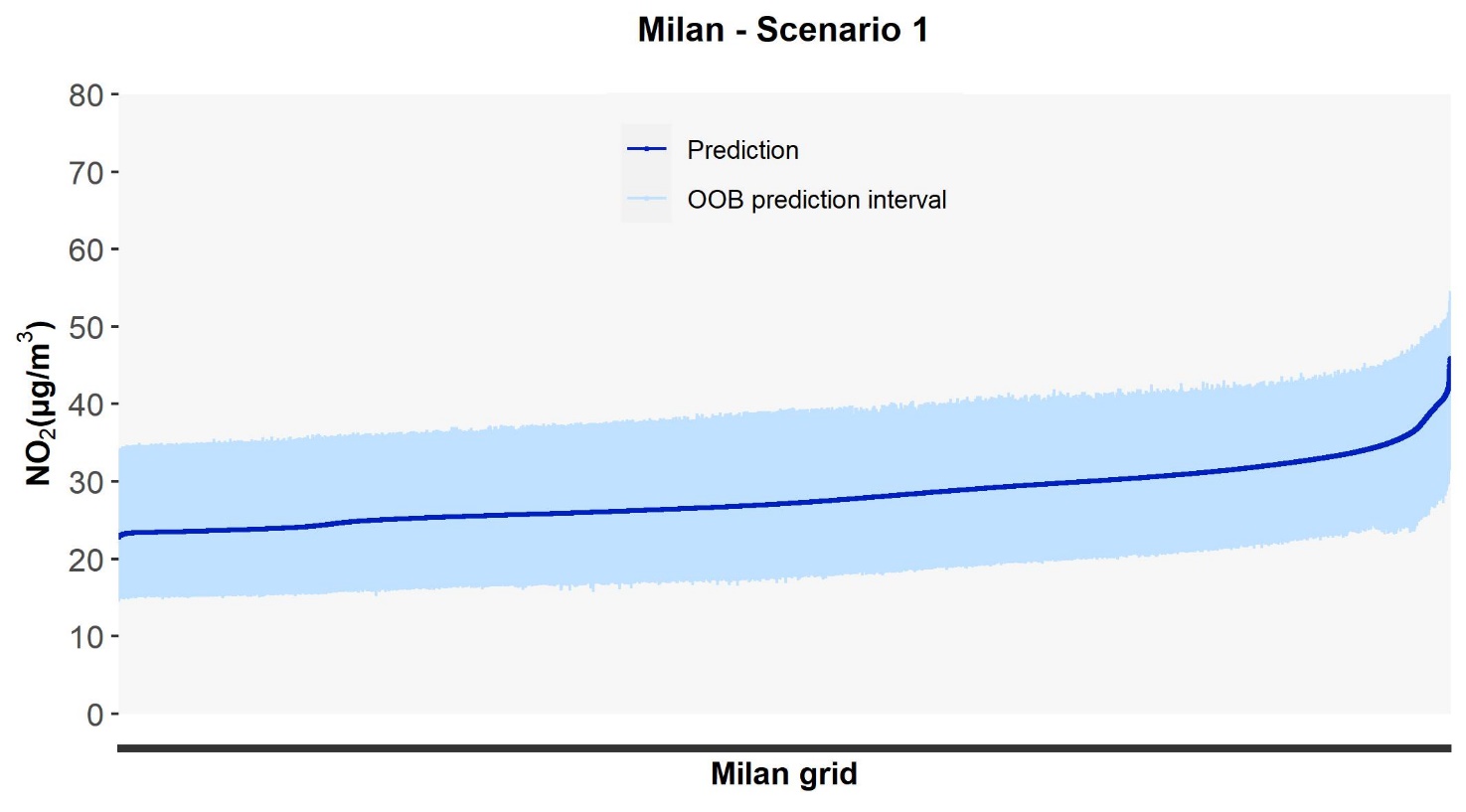


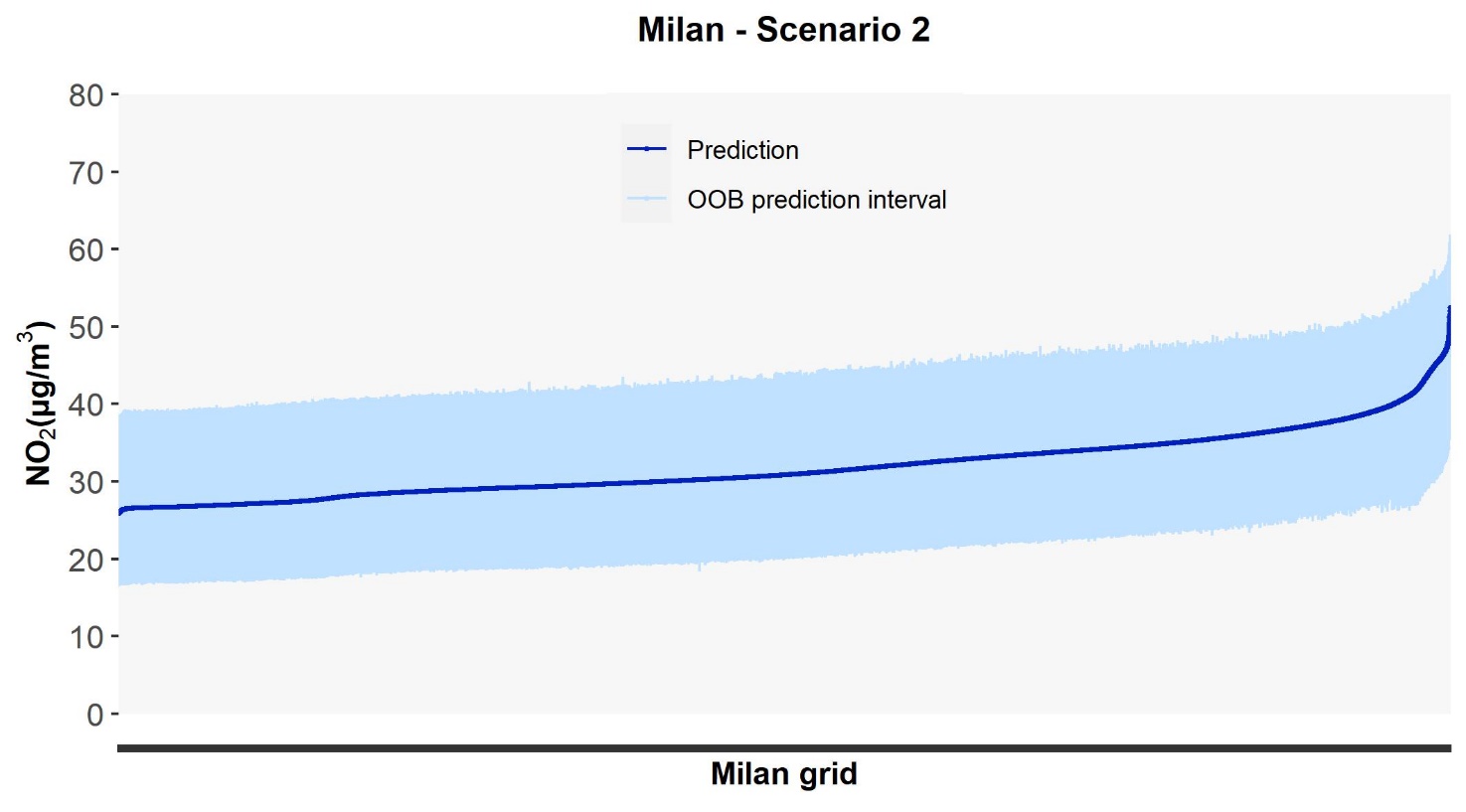


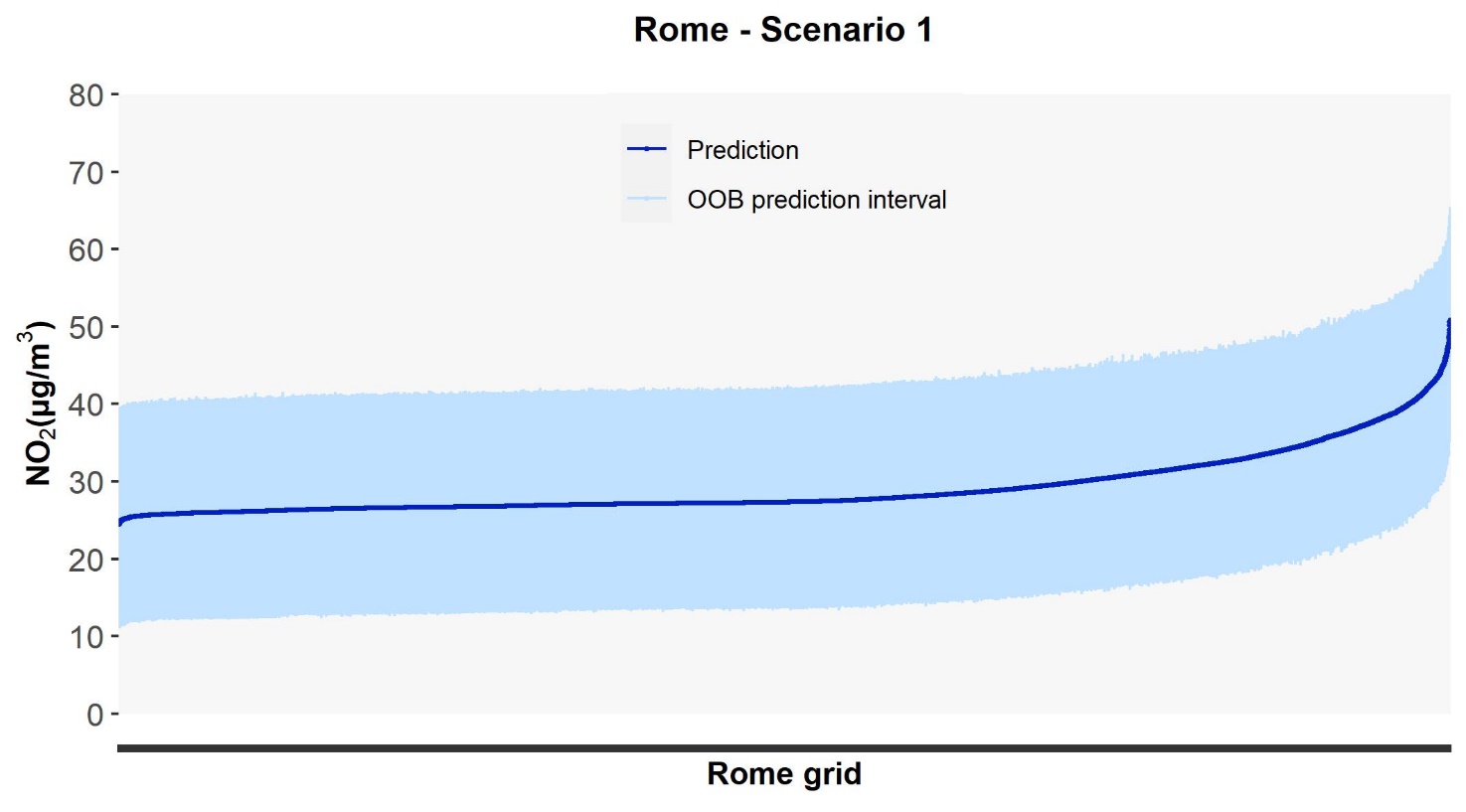


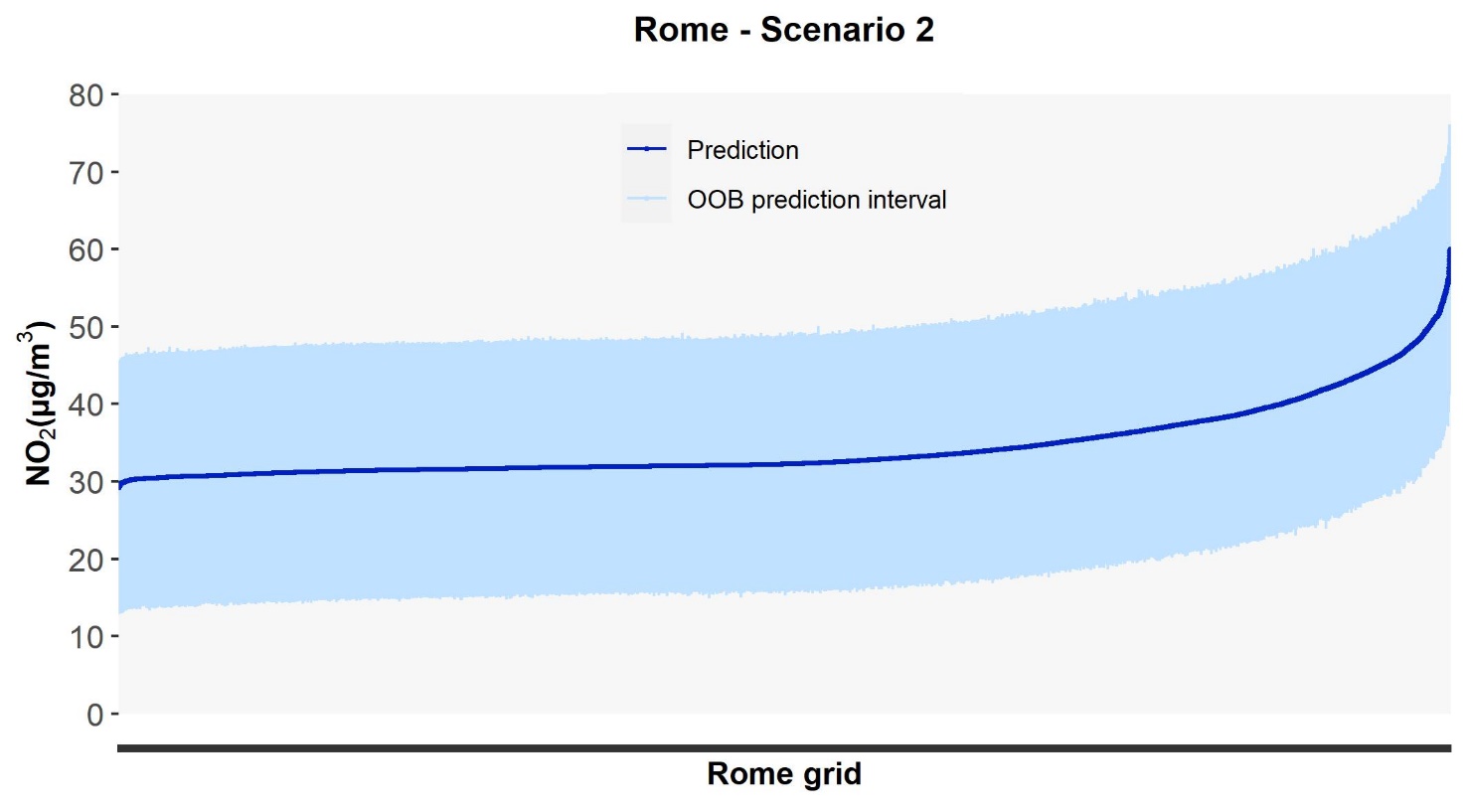


**A.9** NO_2_ attributable deaths (95% CI*) according to the uncertainties of the different stages of the analysis.

|  |  | **Milan** | | **Rome** | |
| --- | --- | --- | --- | --- | --- |
|  |  | **Scenario 1** | **Scenario 2** | **Scenario 1** | **Scenario 2** |
| **Point estimate** | | 1130 | 1343 | 2541 | 3145 |
|  |  | (583-1891) | (695-2234) | (1315-4230) | (1637-5192) |
| **Annual adjustment factor** | **Min** | 1330 | 1535 | 2975 | 3590 |
|  |  | (688-2213) | (798-2532) | (1546-4922) | (1877-5890) |
|  | **Max** | 995 | 1184 | 2199 | 2733 |
|  |  | (511-1670) | (611-1978) | (1134-3679) | (1417-4538) |
| **OOB prediction interval**  **for the RF estimates** | **Lower** | / | 1207 | / | 2788 |
|  |  |  | (623-2016) |  | (1446-4526) |
|  | **Upper** | / | 1504 | / | 3544 |
|  |  |  | (781-2490) |  | (1852-5817) |
| **OOB prediction interval**  **for the LURF estimates** | **Lower** | 653 | 804 | 1167 | 1543 |
|  |  | (334-1107) | (412-1357) | (596-1978) | (792-2602) |
|  | **Upper** | 1603 | 1873 | 3855 | 4683 |
|  |  | (834-2648) | (980-3070) | (2020-6303) | (2475-7566) |

* RR = 1.04 (95% CI 1.02 - 1.07) (HEI, 2021)

**A.10** District administrative boarders for Milan (A) and Rome (B).


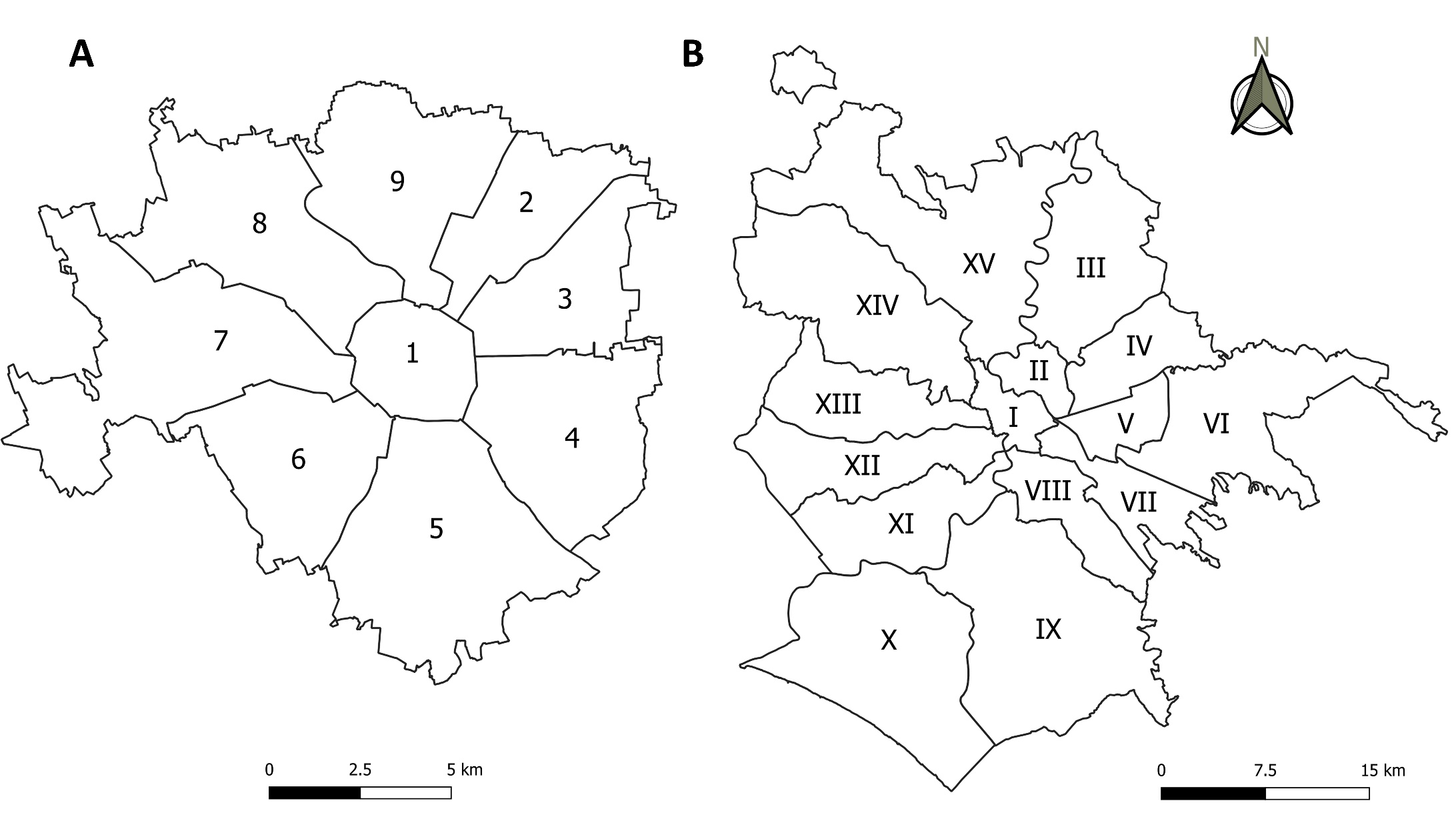


**A.11** NO_2_ attributable deaths, NO_2_ attributable mortality rates (x 10.000 inhabitants) and percentage change for each district of Milan.

| **District** | **Area (Km^2^)** | **Population** | **Deaths** | **SCENARIO 1** | | | **SCENARIO 2** | | | **Attributable mortality rate increment  (S2 VS S1)** |
| --- | --- | --- | --- | --- | --- | --- | --- | --- | --- | --- |
|  |  |  |  | **Median NO_2_ concentration (p5-p95) (µg/m^3^)** | **NO_2_ attributable deaths** | **NO_2_ attributable mortality rate (x10.000)** | **Median NO_2_ concentration (p5-p95) (µg/m^3^)** | **NO_2_ attributable deaths** | **NO_2_ attributable mortality rate (x10.000)** |  |
| 1 | 9.4 | 97728 | 975 | 31.6 (29.4-37.0) | 84 | 8.56 | 35.9 (33.4-42.2) | 99 | 10.13 | 18.3% |
| 2 | 12.6 | 163376 | 1210 | 30.6 (27.6-37.6) | 102 | 6.25 | 34.8 (31.5-42.8) | 121 | 7.41 | 18.6% |
| 3 | 14.4 | 145059 | 1418 | 30.4 (25.8-38.4) | 122 | 8.40 | 34.5 (29.5-43.8) | 144 | 9.95 | 18.5% |
| 4 | 20.7 | 161922 | 1750 | 29.3 (24.8-36.6) | 144 | 8.89 | 33.3 (28.1-41.6) | 171 | 10.56 | 18.8% |
| 5 | 29.9 | 128039 | 1478 | 26.0 (23.5-33.7) | 118 | 9.18 | 29.6 (26.8-38.3) | 140 | 10.92 | 19.0% |
| 6 | 18.3 | 151774 | 1723 | 29.6 (25.0-35.1) | 137 | 9.05 | 33.6 (28.3-39.9) | 163 | 10.75 | 18.8% |
| 7 | 31.4 | 173373 | 1789 | 27.1 (23.6-35.5) | 142 | 8.16 | 31.0 (26.9-40.3) | 168 | 9.71 | 19.0% |
| 8 | 23.9 | 192965 | 1878 | 29.9 (25.6-36.7) | 150 | 7.80 | 33.9 (29.1-41.8) | 179 | 9.28 | 19.0% |
| 9 | 21.0 | 181654 | 1641 | 29.9 (25.9-36.1) | 132 | 7.29 | 34.0 (29.5-41.0) | 158 | 8.68 | 19.1% |

**A.12** NO_2_ attributable deaths, NO_2_ attributable mortality rates (x 10.000 inhabitants) and percentage change for each district of Rome.

| **District** | **Area (Km^2^)** | **Population** | **Deaths** | **SCENARIO 1** | | | **SCENARIO 2** | | | **Attributable mortality rate increment  (S2 VS S1)** |
| --- | --- | --- | --- | --- | --- | --- | --- | --- | --- | --- |
|  |  |  |  | **Median NO_2_ concentration (p5-p95) (µg/m^3^)** | **NO_2_ attributable deaths** | **NO_2_ attributable mortality rate (x10.000)** | **Median NO_2_ concentration (p5-p95) (µg/m^3^)** | **NO_2_ attributable deaths** | **NO_2_ attributable mortality rate (x10.000)** |  |
| **I** | 20.1 | 180124 | 1719 | 38.4 (30.9-45.8) | 187 | 10.36 | 45.3 (36.4-54.4) | 229 | 12.70 | 22.6% |
| **II** | 19.7 | 168357 | 1755 | 36.5 (28.1-45.7) | 181 | 10.74 | 43.0 (33.1-54.0) | 222 | 13.20 | 22.8% |
| **III** | 98.0 | 205341 | 2191 | 27.3 (26.4-37.1) | 202 | 9.86 | 32.1 (31.1-43.8) | 249 | 12.15 | 23.2% |
| **IV** | 49.0 | 176660 | 1705 | 30.6 (26.9-41.2) | 158 | 8.97 | 36.1 (31.9-48.4) | 196 | 11.10 | 23.7% |
| **V** | 27.0 | 247697 | 2639 | 33.2 (27.9-42.9) | 260 | 10.49 | 39.2 (33.0-50.8) | 321 | 12.96 | 23.6% |
| **VI** | 114.0 | 258902 | 1943 | 28.0 (26.5-37.2) | 157 | 6.06 | 33.2 (31.4-43.9) | 196 | 7.58 | 25.0% |
| **VII** | 45.5 | 307645 | 3280 | 31.8 (26.7-42.5) | 325 | 10.58 | 37.4 (31.7-50.4) | 402 | 13.05 | 23.4% |
| **VIII** | 47.2 | 130978 | 1491 | 29.2 (26.4-39.8) | 142 | 10.83 | 34.3 (31.3-47.3) | 175 | 13.34 | 23.2% |
| **IX** | 183.5 | 182261 | 1536 | 27.2 (26.0-36.8) | 126 | 6.90 | 32.0 (30.9-43.2) | 157 | 8.60 | 24.6% |
| **X** | 151.0 | 231598 | 1884 | 26.9 (25.5-33.5) | 151 | 6.51 | 31.9 (30.3-39.6) | 189 | 8.17 | 25.5% |
| **XI** | 71.5 | 155989 | 1484 | 28.4 (26.5-38.6) | 138 | 8.82 | 33.5 (31.4-45.5) | 170 | 10.93 | 24.0% |
| **XII** | 73.1 | 140996 | 1521 | 27.4 (26.3-37.9) | 138 | 9.82 | 32.3 (30.9-44.9) | 172 | 12.18 | 24.0% |
| **XIII** | 67.1 | 133478 | 1340 | 27.4 (26.0-38.7) | 124 | 9.32 | 32.2 (30.6-45.5) | 154 | 11.50 | 23.5% |
| **XIV** | 133.5 | 192173 | 1795 | 27.2 (25.7-34.4) | 153 | 7.98 | 32.0 (30.5-40.6) | 191 | 9.92 | 24.4% |
| **XV** | 187.4 | 160601 | 1236 | 27.1 (26.0-33.9) | 98 | 6.12 | 31.9 (30.7-39.8) | 123 | 7.65 | 25.0% |
